# Exosomes in Epilepsy of Tuberous Sclerosis Complex: Carriers of Pro-Inflammatory MicroRNAs

**DOI:** 10.3390/ncrna7030040

**Published:** 2021-07-10

**Authors:** Daniela Cukovic, Shruti Bagla, Dylan Ukasik, Paul M. Stemmer, Bhanu P. Jena, Akshata R. Naik, Sandeep Sood, Eishi Asano, Aimee Luat, Diane C. Chugani, Alan A. Dombkowski

**Affiliations:** 1 Department of Pediatrics, School of Medicine, Wayne State University, Detroit, MI 48201, USA; dcukovi@med.wayne.edu (D.C.); sbagla@med.wayne.edu (S.B.); easano@med.wayne.edu (E.A.); 2 Translational Neurosciences Program, Wayne State University, Detroit, MI 48201, USA; gw4458@wayne.edu; 3 Institute of Environmental Health Sciences, Wayne State University, Detroit, MI 48201, USA; pmstemmer@wayne.edu; 4 Department of Physiology, School of Medicine, Wayne State University, Detroit, MI 48201, USA; bjena@med.wayne.edu (B.P.J.); anaik@med.wayne.edu (A.R.N.); 5 Department of Neurosurgery, School of Medicine, Wayne State University, Detroit, MI 48201, USA; ssood@med.wayne.edu; 6 Department of Neurology, School of Medicine, Wayne State University, Detroit, MI 48201, USA; luat1al@cmich.edu; 7 Department of Pediatrics, Central Michigan University, Mt Pleasant, MI 48858, USA; 8 Departments of Communication Sciences and Disorders, and Chemistry and Biochemistry, University of Delaware, Newark, DE 19713, USA; dchugani@udel.edu

**Keywords:** microRNA, exosome, epilepsy, tuberous sclerosis complex, neuroinflammation, toll-like receptor

## Abstract

Exosomes are a class of small, secreted extracellular vesicles (EV) that have recently gained considerable attention for their role in normal cellular function, disease processes and potential as biomarkers. Exosomes serve as intercellular messengers and carry molecular cargo that can alter gene expression and the phenotype of recipient cells. Here, we investigated alterations of microRNA cargo in exosomes secreted by epileptogenic tissue in tuberous sclerosis complex (TSC), a multi-system genetic disorder that includes brain lesions known as tubers. Approximately 90% of TSC patients suffer from seizures that originate from tubers, and ~60% are resistant to antiseizure drugs. It is unknown why some tubers cause seizures while others do not, and the molecular basis of drug-resistant epilepsy is not well understood. It is believed that neuroinflammation is involved, and characterization of this mechanism may be key to disrupting the “vicious cycle” between seizures, neuroinflammation, and increased seizure susceptibility. We isolated exosomes from epileptogenic and non-epileptogenic TSC tubers, and we identified differences in their microRNA cargo using small RNA-seq. We identified 12 microRNAs (including miR-142-3p, miR-223-3p and miR-21-5p) that are significantly increased in epileptogenic tubers and contain nucleic acid motifs that activate toll-like receptors (TLR7/8), initiating a neuroinflammatory cascade. Exosomes from epileptogenic tissue caused induction of key pathways in cultured cells, including innate immune signaling (TLR), inflammatory response and key signaling nodes *SQSTM1* (p62) and *CDKN1A* (p21). Genes induced in vitro were also significantly upregulated in epileptogenic tissue. These results provide new evidence on the role of exosomes and non-coding RNA cargo in the neuroinflammatory cascade of epilepsy and may help advance the development of novel biomarkers and therapeutic approaches for the treatment of drug-resistant epilepsy.

## 1. Introduction

The critical role of microRNAs in neurological development and function is becoming increasingly evident, and dysregulation of microRNAs has been implicated in a range of neurological disorders. Altered microRNA levels have been reported in numerous epilepsy studies [1]. We previously identified several overexpressed microRNAs that are a distinctive signature of epileptogenic tissue in patients with tuberous sclerosis complex (TSC), and increased levels coincided with a clinical imaging biomarker of neuroinflammation [2]. TSC is a multisystem genetic disorder that arises from mutations in the *TSC1* and *TSC2* genes, which lead to hyperactivation of the mTOR pathway. TSC typically involves hamartomatous lesions in a range of organs, including the brain. A common clinical feature of TSC is the presence of lesions in the cerebral cortex known as tubers. TSC tubers are associated with significant clinical manifestations, including epilepsy. Approximately 90% of TSC patients suffer from seizures, and many have drug-resistant epilepsy. An open question is why some tubers cause seizures while others do not. It is believed that neuroinflammation contributes to TSC epilepsy; but this mechanism is not well characterized. It is thought that a “vicious cycle” of seizure activity and neuroinflammation exists in drug-resistant epilepsy, where an initial insult (genetic, trauma, etc.) initiates seizures, which then induce neuroinflammation that lowers seizure thresholds and further increases seizure susceptibility [3,4]. The link between neuroinflammation and seizures is supported by evidence from molecular studies of epileptogenic human tissue, as well as animal studies that demonstrate pro-inflammatory cytokine induction results in increased seizure susceptibility. Further study of the relationship between neuroinflammation and seizures is essential for the development of improved therapeutics for treatment of drug-resistant epilepsy.

Notable among elevated microRNAs that we previously identified in epileptogenic TSC tissue is miR-142-3p [2]. We found a strong correlation between tissue levels of miR-142-3p and a clinical imaging marker of neuroinflammation (alpha[C-11]methyl-L-tryptophan positron emission tomography, AMT-PET), but the mechanistic linkage was unclear. The canonical role of microRNAs as post-transcriptional repressors is widely recognized, but recent work has illuminated another role for some small, non-coding RNAs as ligands and activators of innate immune system toll-like receptors 7 and 8. This pathway induces a pro-inflammatory signaling cascade. Another research group determined that miR-142-3p is a ligand and activator of toll-like receptor 7 (TLR7), which initiates a neuroinflammatory cascade [5]. Indeed, we subsequently found activated TLR7 corresponding to miR-142-3p levels in epileptogenic tissue [6]. TLR7 and closely related TLR8 are receptors of the innate immune system that serve as detectors of pathogen- and damage-associated molecular patterns in single-stranded RNA. Activation of these receptors by exogenous (e.g., viral) RNA alerts the immune system and initiates an inflammatory cascade. TLR7/8 can also be activated through the binding of endogenous small RNAs having G/U-rich sequence motifs, including miR-142-3p, miR-21-5p, miR-29a and let-7 [7,8]. Recent work has revealed that TLRs play a prominent role in neuroinflammation, and injured neurons can release small RNAs that activate TLR7/8 [9,10]. Immune and inflammatory signaling downstream of TLR activation results in activation of NF-κB and production of pro-inflammatory cytokines. Pro-inflammatory cytokines can lower seizure thresholds and increase seizure susceptibility [11,12].

Recent reports on extracellular vesicles (EV) and their microRNA cargo led us to hypothesize that our observed TLR7 activation in TSC epilepsy may be mediated by exosomes. Exosomes are small, membrane-bound EV that transport protein, RNA, DNA and lipids between cells. Exosome trafficking is an important intercellular signaling mechanism, yet the role in cellular function and disease is only starting to be uncovered. Exosome cargo is often enriched with microRNAs, which can alter target gene expression and the phenotype of recipient cells. Some microRNAs carried by exosomes have been shown to activate TLR7/8 by acting as ligands of the receptors [7]. Given our previous observation of activated TLR7 in epileptogenic TSC tissue, we sought to determine if this neuroinflammatory signaling is mediated by exosomes. In this study we isolated exosomes from TSC tubers that were surgically resected for the treatment of drug-resistant epilepsy. We then used RNA-seq to quantify the abundance of exosomal microRNAs and compared microRNA levels in exosomes from epileptogenic and non-epileptogenic tissues. Based on our previous work that linked miR-142-3p to TLR7 activation and neuroinflammation in epileptogenic lesions, we focused our analysis on exosomal microRNAs having TLR7/8 activating motifs. We found significant enrichment of microRNAs having TLR7/8 activation motifs in exosomes from epileptogenic tubers. We used an in vitro model to determine the effect on gene expression in cells incubated with exosomes extracted from epileptogenic tissue. The analysis of altered genes identified key signaling pathways and nodes. The signature of exosome-induced gene expression was also found in epileptogenic TSC tissue.

## 2. Results

### 2.1. Isolation and Characterization of Exosome Enriched Fractions of Extracellular Vesicles (EV) from Brain Tissue

**Figure 1:**
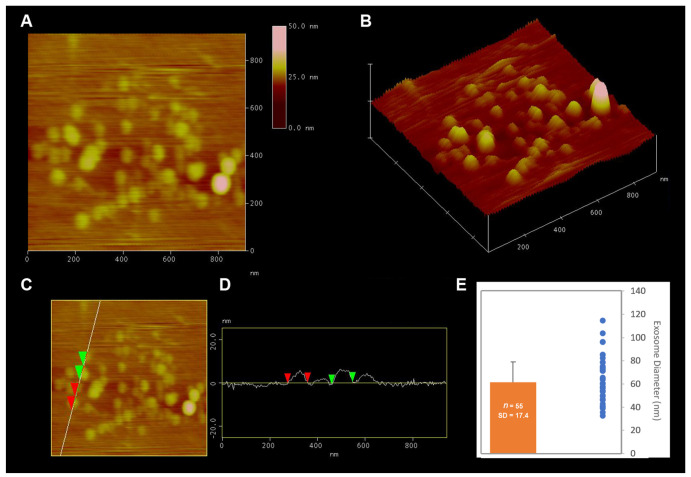
Atomic force micrograph of isolated exosomes demonstrates a distribution of 30–115 nm and an average size of 60 nm. (**A**) AFM 2-D micrograph of isolated exosomes placed on mica. (**B**) AFM 3-D micrograph of the isolated exosomes in A. (**C**,**D**) Cross-section analysis of exosomes, and (**E**) Bar graph of exosome size and the size distributions to the right as dots.

Using a method previously validated for brain tissue, we isolated EV from cortical tubers that were surgically resected from pediatric patients for the treatment of drug-resistant epilepsy [13]. Extracellular vesicles were isolated from three epileptogenic (seizure onset) tubers and three non-epileptogenic (non-onset) tubers. Isolation of exosomes from solid tissue currently requires a substantial amount of tissue, and specimens from epilepsy surgery are very limited, typically small, and difficult to obtain. We anticipate that future improvements in EV isolation methods will expand the number of samples available for studies of exosomes in human epilepsy. The epileptogenic categories of our samples were determined by electrocorticography (ECoG) during surgery. We used atomic force microscopy (AFM) to examine the size and morphology of isolated EVs. AFM revealed spherical vesicles of 30–115 nm, with an average diameter of 60 nm (Figure 1A–E). These results are consistent with accepted exosome features [14]. AFM also revealed that the isolated vesicles were free of contaminating debris. To evaluate the protein composition of isolated vesicles we used LC-MS/MS proteomics to identify proteins in a representative sample of isolated EV. We identified 652 proteins with a false discovery rate less than 10%. To determine if the protein content is consistent with known EV cargo, we compared the identified proteins to the top 50 proteins most frequently reported in EV, per the Vesiclepedia database [15]. We found that 42 of the top 50 EV proteins are present, including the established exosome marker CD81. Syntenin-1 was also present and is considered a distinct marker that is specific to “bona fide” exosomes [16]. The results demonstrate that our isolated vesicles have morphological and molecular features that are characteristic of exosomes.

### 2.2. RNA-Seq Analysis Reveals Enrichment of TLR7/8-Activating MicroRNAs in Epileptogenic Exosomes

**Figure 2:**
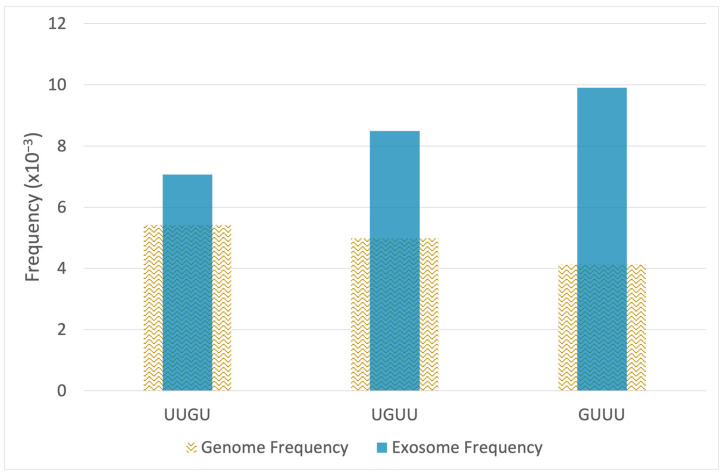
Exosomes isolated from epileptogenic tissue are enriched with microRNAs having G/U-rich motifs that activate TLR7/8. The sequences of 37 microRNAs increased in the cargo of epileptogenic exosomes were analyzed for nucleic acid motifs that function as ligands and activators of TLR7/8. The frequency of TLR7/8 motifs is significantly higher in the set of 37 exosomal microRNAs compared to the overall set of human microRNAs (*p* = 0.017, hypergeometric test).

Small RNA-seq was used to quantify and compare microRNA levels in EV of the epileptogenic and non-epileptogenic groups. Thirty-seven microRNAs had a statistically significant increase in epileptogenic EV (*p* ≤ 0.05) and a minimum 1.5-fold change. We analyzed the sequences of the 37 microRNAs to identify known TLR7/8-activating motifs. Using the top 3 G/U-rich motifs (UUGU, GUUU, UGUU) having the strongest propensity to activate TLR7/8 [17], we tabulated TLR7/8-activating motifs found in each microRNA. This was accomplished by extracting each 4-mer nucleotide in a sliding window across each microRNA sequence and matching it to the three motifs. Of the 37 microRNAs increased in epileptogenic EV, 12 had one or more TLR7/8-activating motifs (Table 1). These microRNAs had a mean increase of 4-fold. In this set, a total of 18 motifs were identified out of a total of 707 4-mers extracted from the 37 increased microRNAs. To determine the significance of this finding, we compared this result to the number of motifs found in the entire set of all human microRNAs. We downloaded 2656 mature human microRNA sequences from miRbase and performed the same motif tabulation, finding a total of 718 TLR7/8 motifs out of a total of 49,418 4-mers extracted from the background set of 2656 microRNA sequences. Analysis of these observed frequencies using the hypergeometric test reveals that the overall set of 37 EV microRNAs is significantly enriched for TLR7/8 motifs (*p* = 0.017), which are found in the subset of 12 microRNAs shown in Table 1 [18]. Figure 2 shows frequencies of each motif in the EV enriched microRNAs compared to the genome-wide set of microRNAs.

### 2.3. Exosomes from Epileptogenic Tissue Induce Key Signaling Pathways in Recipient Cells

**Figure 3:**
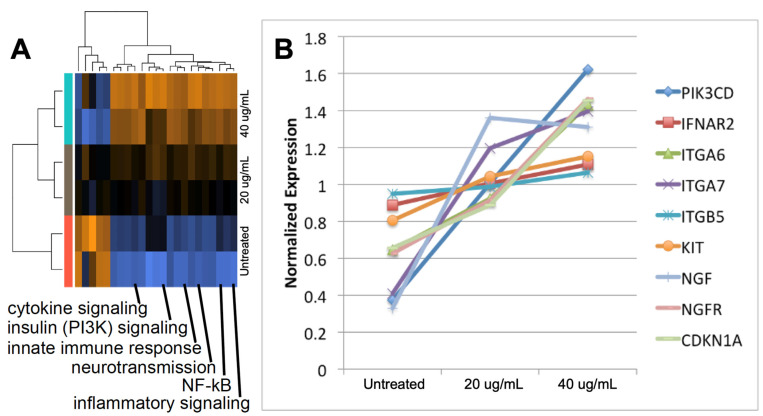
Inflammatory signaling is induced in SH-SY5Y cells incubated with 2 doses of exosomes isolated from epileptogenic tuber tissue. Gene expression analysis was performed using the Nanostring nCounter Neuroinflammation panel of 770 genes. (**A**) Induced key pathways included innate immune response (TLR), cytokine signaling and NF-κB. Orange indicates upregulation of pathway genes; blue indicates lower expression. (**B**) Genes in the PI3K/AKT pathway are induced in a dose-response manner.

We investigated changes in gene expression in cells incubated in culture with exosomes isolated from epileptogenic tissue. We used SH-SY5Y cells, which have been widely used as a neuronal model [19] and are known to express TLR7/8 [20,21]. The cells were treated with 2 doses of exosomes from epileptogenic tuber tissue (20 and 40 ug/mL) and compared to untreated control cells. We performed gene expression analysis using the NanoString nCounter Neuroinflammation Panel [22]. This panel includes probes for 770 genes involved in key pathways representing inflammation, immunity, neurobiology, metabolism, and stress. The nCounter technology enables direct measurement of transcript abundance and provides quantitative sensitivity comparable to qRT-PCR, while avoiding bias inherent to reverse transcription and amplification reactions. Pathway analysis using NanoString nSolver software revealed a dose-response pattern of pathway alterations (Figure 3A). A cluster of pathways exhibited a dose-dependent increase, including inflammation, innate immune response (TLR), cytokine signaling, NF-κB, insulin (PI3K) signaling, and apoptosis. Induction of these pathways are characteristic of TLR7/8 activation.

We performed statistical analysis to identify differentially expressed (DE) genes, comparing the 40 ug/mL exosome dose to untreated controls. We identified 105 DE genes (*p* ≤ 0.05), with 84 increased and 21 repressed. The upregulated genes included established markers of TLR7/8 activation that are pro-inflammatory: *TRADD* (3.9 fold, *p* = 0.027), *CD83* (1.9 fold, *p* = 0.031), and *CASP7* (1.8 fold, *p* = 0.002) [23]. Using DAVID for functional annotation analysis, we identified 9 genes in the PI3K/AKT pathway induced in SH-SY5Y cells exposed to exosomes from epileptogenic tissue, and these genes exhibited a dose response pattern (Figure 3B). The activated pathways and key nodes are consistent with previous reports of pathway activation in epileptogenic TSC tissue. The PI3K/AKT pathway is immediately upstream of the mTOR pathway. Activation of AKT leads to disruption of the hamartin/tuberin complex (TSC1/TSC2) and may contribute to hyperactivation of mTOR [24].

**Figure 4:**
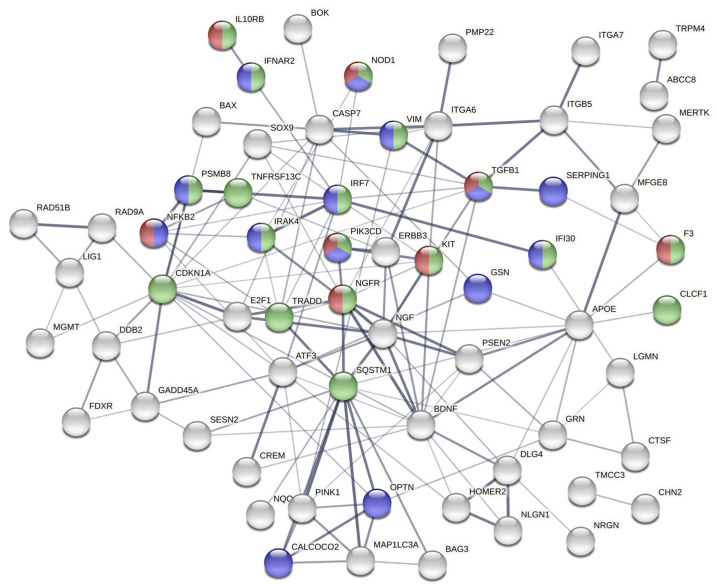
The network of genes induced by epileptogenic exosomes is highly connected and converges on several key pathways. Genes involved in innate immune response (*p* = 4.81 × 10^−5^), inflammatory response (*p* = 0.0035), and cytokine mediated signaling (*p* = 9.02 × 10^−10^) colored as blue, red, and green, respectively. The most highly connected nodes include *SQSTM1* (p62) and *CDKN1A* (p21).

**Figure 5:**
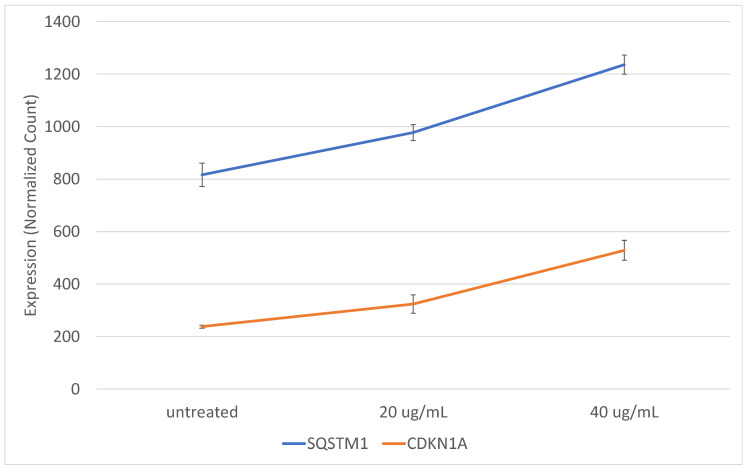
Key signaling nodes are induced by exosomes from epileptogenic tissue. SH-SY5Y cells were incubated with exosomes from epileptogenic tissue at 20 and 40 ug/mL. Gene expression was measured using Nanostring nCounter assays. *SQSTM1* and *CDKN1A* are highly connected nodes in the network of induced genes and have a clear dose response. Error bars represent standard error of the mean.

Highly interconnected hubs in protein-protein interaction (PPI) networks are characteristic of functionally significant proteins. We identified the PPI network among the set of 84 induced genes using the STRING server [25]. The PPI network of this gene set is significantly enriched for interactions compared to what is expected by chance (*p* < 1.0 × 10^−16^). Interconnected genes (64) are shown in Figure 4, with genes involved in innate immune response (*p* = 4.81 × 10^−5^), inflammatory response (*p* = 0.0035), and cytokine mediated signaling (*p* = 9.02 × 10^−10^) colored as blue, red, and green, respectively. Fourteen of the induced genes are involved in innate immune response (blue), and several of these are critical signaling mediators immediately downstream of TLR7/8 (*IRF7, IRAK4, NFKB2*) [26,27]. Highly connected key nodes in the network were identified by tabulating the number of interactions for each node. The top nodes with the greatest number of interactions include *CDKN1A* (p21), and *SQSTM1* (p62). *SQSTM1* (sequestosome-1, p62) is involved in immune system signaling, NF-κB activation, autophagy, and nutrient-dependent activation of mTOR [28]. A dose response is evident in the gene expression patterns of *SQSTM1* and *CDKN1A* in cells treated with exosomes from epileptogenic tissue (Figure 5).

To investigate if the exosome microRNAs may be exerting a repressive effect on genes in innate immune signaling and inflammatory pathways via canonical microRNA-mediated post-transcriptional repression, we performed microRNA target prediction analysis of the 37 upregulated microRNAs found in the epileptogenic exosomes. We used the well-established miRWalk consensus prediction tool [29] and identified 142 high confidence target genes. We then performed a gene ontology analysis of enriched biological processes among this gene set using the STRING server. Innate immune signaling and inflammation were not among the enriched processes. Additionally, none of the predicted targets were among the 21 genes repressed in the SH-SY5Y cells treated with epileptogenic exosomes. These results indicate that the exosome microRNAs are activating innate immune signaling and neuroinflammatory pathways as ligands of the TLR7/8 receptors, but they do not have an overall repressive effect on these pathways as inhibitors of gene transcription.

### 2.4. The Gene Expression Signature Induced In Vitro by Epileptogenic Exosomes Is Also Found in Epileptogenic Tissue

**Figure 6:**
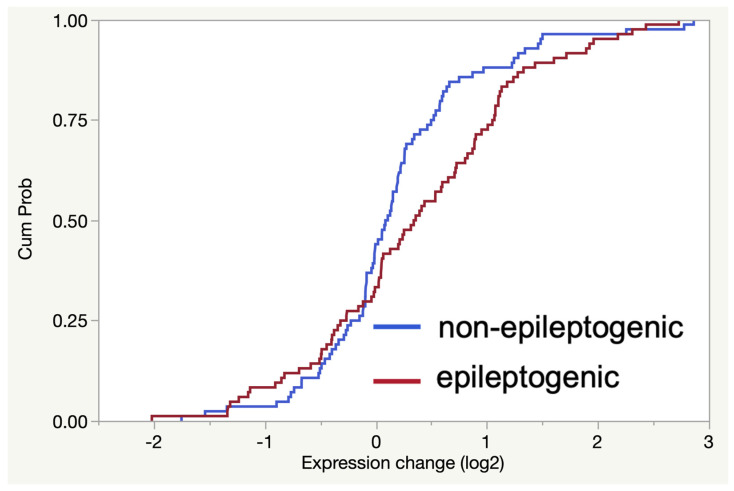
The overall set of 84 genes induced by epileptogenic exosomes in vitro is also increased in epileptogenic TSC tubers. The fold change (log2) for each gene was calculated with respect to normal control tissue and the cumulative distribution function (CDF) for the 84 genes was plotted for epileptogenic (red) and non-epileptogenic (blue) tubers. The expression of the gene set is significantly increased in epileptogenic tubers (*p* = 0.017, Kolmogorov-Smirnov test), as indicated by the rightward shift of the distribution.

Using the expression signature of 84 genes induced by epileptogenic exosomes in vitro, we investigated expression levels of the same gene set in surgically resected TSC tubers. We used the Nanostring gene expression assay and compared expression changes in epileptogenic (5) and non-epileptogenic tubers (5). Fold changes were calculated for the 84 genes in each group, using non-tuber tissue (3) as a control reference. We found that the collective set of 84 genes is significantly induced in epileptogenic tubers compared to non-epileptogenic tissue (Figure 6, *p* = 0.017). These results demonstrate that the gene expression pattern induced by epileptogenic exosomes in vitro is also evident in epileptogenic tissue.

## 3. Discussion

In this study we isolated extracellular vesicles from brain tissue that was surgically resected to treat drug-resistant epilepsy in TSC patients. Analysis of isolated vesicles using atomic force microscopy and LC-MS/MS proteomics demonstrated that morphological and molecular features of isolated EV are consistent with exosomes. We performed small RNA-seq on isolated EV and compared microRNA levels between EV from epileptogenic and non-epileptogenic TSC tubers. We found significant enrichment in epileptogenic exosomes for microRNAs having nucleic acid motifs that activate TLR7/8. Among the microRNAs that were increased and identified as having TLR7/8 motifs were miR-142-3p and miR-223-3p. We previously reported that expression levels of these two microRNAs were highly correlated and markedly increased in tissue lysates from epileptogenic TSC tubers that were clinically identified with a marker of neuroinflammation (AMT-PET), although the mechanism of association between these two microRNAs and neuroinflammation was not clear [2]. The results of our current investigation demonstrate that these microRNAs and others with TLR7/8-activating motifs are significantly increased in the cargo of exosomes of epileptogenic tissue, thus providing evidence of a neuroinflammatory signaling mechanism mediated by extracellular vesicles. The set of increased microRNAs also includes miR-21-5p, which has also been confirmed to activate TLR7/8 [30,31].

We incubated a neuronal cell model (SH-SY5Y) with exosomes from epileptogenic tissue and found induction of several key pathways. Our network and pathway analysis revealed induction of numerous genes involved in innate immune signaling (TLR), inflammatory response and cytokine signaling. Highly connected key nodes in these pathways include *SQSTM1* (p62) and *CDKN1A* (p21). *CDKN1A* was previously reported as significantly increased in TSC tubers and linked to immune system signaling [32]. An integrative analysis of epilepsy animal models and human epilepsy tissue found that *CDKN1A* is a central hub in epileptogenesis [33]. *SQSTM1* is involved in cytokine signaling of the immune system and is a central regulator for activation of several key signaling pathways, including mTORC1 and NF-κB [28]. *SQSTM1* is an essential hub of nutrient-sensing and regulation of cellular metabolism through mTORC1. Amino acid availability induces *SQSTM1* to interact with mTOR and raptor, stabilizing the mTORC1 complex and results in mTORC1 activation [34]. *SQSTM1* has been found to be overexpressed in several types of cancer, including glioblastoma and melanoma [34,35]. The accumulation of SQSTM1 is believed to be tumorigenic through activation of mTORC1, leading to increased cell proliferation and a decrease in autophagy. Elevated *SQSTM1* was found in kidney and lung lesions of TSC patients [36]. Several studies have shown increased levels of SQSTM1 within TSC tubers and focal cortical dysplasia type 2B [37,38]. Hyperactivated mTORC1 is a hallmark of these malformations of cortical development and may be further exacerbated by SQSTM1 accumulation. Recent work has highlighted considerable crosstalk between the innate immune system and SQSTM1, with a convergence on mTORC1 activation [39,40]. Our results indicate that exosomal signaling in epileptogenic lesions results in increased *SQSTM1*, which may contribute to mTORC1 signaling. This mechanism warrants further study.

We queried proteomics data from our previous study that compared epileptogenic tubers (4) to adjacent non-tuber control tissue (4) and found that SQSTM1 protein was elevated 16% in epileptogenic tubers [41]. The associated mRNA was similarly increased in epileptogenic tubers, as measured with NanoString analysis in the current study. It is important to consider that proteomics and gene expression analyses of bulk tissue homogenates reflect a heterogenous mixture of a variety of cell types. The exosomes in epileptogenic tissue may be secreted and taken up by specific cell types. Thus, the net effect on gene and protein expression in the recipient cells may be obscured in the mixture of cell types of the heterogenous tissue. Subsequent studies to investigate the source and destination cell types of the altered exosomes will be important to expanding our understanding of this intercellular signaling mechanism.

Genes in the PI3K/AKT pathway were also induced in the SH-SY5Y cells. This pathway is immediately upstream of mTOR [42]. AKT represses the ability of the TSC1/TSC2 complex to inhibit activation of mTOR, and these two genes are most frequently found to be mutated in TSC. Loss of *TSC1* or *TSC2* results in hyperactivation of mTOR, but mutations found in TSC brain tubers are typically heterozygous, leading to an ongoing search for an elusive “second hit”. The convergent effect of exosome-induced *SQSTM1* and PI3K/AKT activation on mTORC1 may contribute to the pathway activation in epileptogenic tubers.

Intercellular signaling by exosomal microRNAs has been implicated in neuroinflammation, and in this study we provide new evidence of this signaling cascade in drug-resistant epilepsy [43]. Our results provide the foundation for subsequent investigations of neuroinflammatory mechanisms in drug-resistant epilepsy and may ultimately illuminate new therapeutic targets and offer novel biomarkers. Exosomes are stable, circulate systemically, and cross the blood-brain barrier (BBB). Importantly, EV transport across the BBB appears to be further facilitated by neuroinflammation [44]. Exosomes originating in epileptogenic tissue may provide a means for non-invasive monitoring of seizure susceptibility and response to treatment.

## 4. Materials and Methods

### 4.1. Brain Tissue Used for Isolation of Extracellular Vesicles

Our study is an analysis of brain tissue that was surgically resected to treat drug-resistant epilepsy at Children’s Hospital of Michigan, Detroit. All patients had a diagnosis of TSC as defined by standard clinical criteria [45]. Preoperative assessment included clinical evaluation, neuroimaging using magnetic resonance imaging (MRI) and AMT-PET [46], and ictal/interictal EEG. Two tissue types were analyzed: epileptogenic tuber tissue characterized by independent epileptiform activity (seizure onset); and non-epileptogenic tuber (non-onset), as determined by electrocorticography (ECoG) [47]. Resected tissue was fresh-frozen for subsequent analysis. All protocols were approved by the Human Investigation Committee at Wayne State University.

### 4.2. Isolation of Extracellular Vesicles from Brain Tissue

To isolate exosome-enriched fractions of extracellular vesicles, we used a method previously validated for brain tissue: PRotein Organic Solvent PRecipitation (PROSPR), with a slight adaptation [13]. Brain tissue was retrieved from a −80 °C archive and kept on dry ice while handling, with minimal exposure at room temperature (RT) during dissection. Each sample was cut into 4 pieces of ~30–40 mg. The pieces were transferred into pre-chilled 2 mL Sample Tubes RB (Qiagen, Valencia, CA, USA), 1 piece per tube plus a stainless steel 5 mm bead. Packed tubes were incubated on dry ice for 15 min and then placed into the insert of a TissueLyser LT Adapter (Qiagen), and incubated for 2 min at RT. At this point, 130 µL of an ice-chilled homogenization buffer was added into each tube, and the TissueLyser LT operated for 4 min at 35 Hz. The homogenization buffer was freshly prepared as a 100 mM ammonium acetate solution supplemented with Protease Inhibitor Cocktail (Sigma–Aldrich, ST. Louis, MO, USA) in dilution 1:100 and kept on ice. Centrifugation was then performed at 15,000× *g*, 4 °C for 10 min. The supernatant was transferred in chilled 1.5 mL tubes. The tubes were kept on ice, while the 2 mL tubes with pellets were placed on dry ice for 15 min. The above comprises the first homogenization cycle. The second cycle of homogenization repeats all the steps described above, and the supernatant was combined with the supernatant from the first homogenization cycle in the same tube and kept on ice. The third and fourth homogenization cycles were the same as the first 2 cycles, except the TissueLyser LT operated for 4 min at 50 Hz.

After homogenization, the volume of the pooled supernatant was approximately 0.5 mL per tube x 4 tubes per sample. The pool of supernatant of each sample was transferred into an ice-chilled 15 mL tube and mixed with chilled acetone (−20 °C) in the ratio 1:4 *v*/*v* supernatant: acetone. The mixture of supernatant and acetone was mixed well with vortex and spun down at 3400 rpm at 4 °C for 1 min. This step pelleted water-soluble proteins and other contaminants, while exosomes were enriched in the acetone supernatant that was aliquoted in 1.5 mL tubes and dehydrated in the SpeedVac Concentrator Savant DNA 120 (Thermo Scientific, Waltham, MA, USA). The whitish-yellowish pellets were stored at −80 °C for subsequent analysis.

### 4.3. Size and Morphology Analysis of Extracellular Vesicles Using Atomic Force Microscopy

Atomic force microscopy (AFM) was performed on isolated exosomes placed on a mica surface in buffer, using a minor modification of our previously published procedure [48,49]. Exosomes were imaged using a Nanoscope IIIa AFM from Digital Instruments. (Santa Barbara, CA, USA). Analysis was performed in the “tapping” mode, using silicon nitride tips with a spring constant of 0.38 N.m^−1^, and an imaging force of <200 pN. Images were obtained in approximately 5 min at line frequencies of 2 Hz, with 512 lines per image, and constant image gains. Topographical dimensions of extracellular vesicles were analyzed using the software nanoscope IIIa4.43r8, supplied by Digital Instruments.

### 4.4. Characterization of EV Proteins Using LC-MS/MS Proteomics

Proteins in the exosome samples were precipitated with 2-volumes of ice-cold 100% methanol-1 mM acetic acid at −20 °C for 1 h. Following centrifugation and removal of the solvent, the air dried pellets were solubilized in 0.5% deoxycholate (DOC), 50 mM TEAB then reduced with 5 mM DTT and alkylated with 15 mM IAA. Excess IAA was quenched with an additional 5 mM DTT. Samples were then digested with sequencing-grade trypsin (Promega) overnight at 37 °C. Digests were acidified with 1% formic acid final then incubated on ice for 30 min. to precipitate the DOC, then supernatants were analyzed by LC-MS/MS. Peptides were separated by reversed-phase chromatography (Acclaim PepMap100 C18 column, Thermo Scientific), followed by ionization with the Nanospray Flex Ion Source (Thermo Scientific), and introduced into a Q Exactive mass spectrometer (Thermo Scientific). Peptides were fragmented with high-energy, collision-induced dissociation (HCD).

Data analysis was performed using Proteome Discoverer 1.4 (Thermo) using the Sequest algorithm (Thermo Fisher). The Uniprot_Hum_Compl_20170714 database was searched for matching sequences and a reverse decoy protein database was run simultaneously for false discovery rate (FDR) determination. Data were searched with a fragment ion mass tolerance of 0.05 Da and a parent ion tolerance of 10 PPM. Carbamidomethylation of cysteine was specified as a fixed modification, deamidation of asparagine and glutamine and oxidation of methionine were specified as variable modifications. Scaffold (version 5.0.0, Proteome Software Inc., Portland, OR, USA) was used to validate MS/MS based peptide and protein identifications. Peptide identifications achieved an FDR less than 1.0% by the Scaffold Local FDR algorithm. Protein identifications were accepted if they could be established at greater than 99.0% probability and contained at least 1 identified peptide. Protein probabilities were assigned by the Protein Prophet algorithm (Nesvizhskii, Al et al. Anal. Chem. 2003;75(17):4646-58). Proteins that contained similar peptides and could not be differentiated based on MS/MS analysis alone were grouped to satisfy the principles of parsimony.

### 4.5. RNA Extraction from Extracellular Vesicles and Tissue

Each exosomal pellet was re-suspended in 100 µL of nuclease-free water, and the suspension was mixed with 700 µL of QIAzol Lysis Reagent (Qiagen) vigorously with vortex, and incubated for 5 min at RT. Then 100 µL of chloroform was added and mixed vigorously for 15 s followed with a 2 min incubation at RT. Separation of the aqueous phase that contained RNA from the organic phase occurred in a centrifuge at 12,000× *g*, 4 °C for 15 min. The aqueous phase was transferred into a new 1.5 mL tube, and 2 volumes of 100% ethanol were added to the tube and mixed with pipetting up and down.

This ethanol mixture was loaded in the volume of 700 µL into the RNeasy MinElute Column (Qiagen) and spun down at 9000× *g* for 30 s at RT. The column loading and spinning were repeated with the remaining ethanol mixture. The column was washed with 500 µL of RWT buffer (initially prepared in 100% isopropanol) and spun down at 9000× *g* for 30 s at RT. The other 2 washing steps used 500 µL of RPE buffer (initially prepared in 100% ethanol) and the same spinning condition. Additional spinning at full speed for 3 min was performed to dry the membrane in the column.

The RNA elution step was undertaken with 20 µL of nuclease-free water that was applied on the membrane, followed with incubation for 2 min at RT and spinning at 10,000× *g* for 1 min. Using the Qubit 2.0 Fluorometer and Qubit miRNA Assay Kit (ThermoFisher/Life Technologies), we measured the miRNA concentration in the exosomal samples. The average yield of small RNA was 6–9 ng per exosomal pellet.

Total RNA was extracted from epileptogenic TSC tissue (5), non-epileptogenic (5) and non-tuber normal controls (3), as reported in [2]. All epileptogenic samples were positive for AMT-PET in clinical evaluation, a marker of inflammation.

### 4.6. RNA-Seq of Exosomal RNA Cargo

Illumina compatible small RNA-seq libraries were prepared using 10 ng of RNA extracted from each EV sample. The Nextflex Small RNA Library Kit from Bioo Scientific was used per the vendor’s guide. A pool of libraries was sequenced by Research Technology Support Facility at Michigan State University using an Illumina sequencer. Quality checks of the prepared library pool was performed using a Qubit dsDNA HS, Advanced Analytical Fragment Analyzer High Sensitivity DNA and Kapa Illumina Library Quantification qPCR assays. The pool was loaded onto one lane of an Illumina HiSeq 4000 flow cell and sequencing was performed in a 1×50 bp single read format using standard HiSeq 4000 SBS reagents. Base calling was conducted by Illumina Real Time Analysis (RTA) v2.7.7 and output of RTA was demultiplexed and converted to FastQ format with Illumina Bcl2fastq v2.19.1. The Galaxy server was used for the trimming and alignment of RNA-seq data. Reads were trimmed with TrimGalore, and short reads were discarded. Trimmed reads were then aligned to hg19 using RNAStar [50]. Feature counts for microRNAs in miRBase were derived using DASHR. Feature counts were normalized for each sample using the total read count for the sample, and then with miR-26a-5p. Several studies have shown miR-26a-5p to be a reliable endogenous control for exosomal microRNAs [51,52,53].

Normalized microRNA counts were log transformed and filtered to exclude low abundance microRNAs. MicroRNAs where the expression count was greater than the 25th percentile for all samples in either group (epileptogenic or non-epileptogenic), were retained for subsequent analysis (309 total). Data analysis procedures were constructed to test the hypothesis that TLR7 activating microRNAs are increased in epileptogenic EV. A one-sided *t*-test was used to calculate *p*-values for microRNA expression differences comparing epileptogenic and non-epileptogenic groups. A *p*-value ≤ 0.05 was specified to indicate statistical significance.

Target prediction of microRNAs was performed using miRWalk with parameters: score ≥ 0.95, 3′ UTR, and consensus prediction from TargetScan, miRDB and miRTarBase [29].

### 4.7. In Vitro Analysis of SH-SY5Y Cell Response to Exosomes

The SH-SY5Y cells (ATCC CRL-2266) were grown with standard media, 1X DMEM/F12 with 10% Hi FBS. A quarter of a million of SH-SY5Y cells were seeded using 24-well plates. The SH-SY5Y cells were incubated with exosomes at 20 ug/mL or 40 ug/mL for 16 h in a cell culture incubator at 37 °C with 5% CO_2_. A very large tuber was required to obtain a sufficient quantity of exosomes for a dose response experiment with replicates. Two replicate wells were used for each dose and media controls. Exosome pellets, previously stored at −80 °C, were re-suspended in 40 µL of 1XPBS (warmed at 37 °C for 10 min.) pipetting up and down several times. Three exosome pellets of the same sample were combined into one tube and incubated in a 37 °C water bath for 5 min. Measured protein concentrations in the exosomal suspensions were acquired using a Qubit 2.0 Fluorometer and Qubit Protein Assay Kit (ThermoFisher/Life Technologies, Waltham, MA, USA). The exosome concentrations, estimated based on protein concentration, were relatively high. Therefore, the volume of 1X PBS with exosomes that was added into the cell culture, was 2–3% of the final media volume. Cells were harvested in 700 µL of QIAzol Lysis Reagent then spun through QIAShredder Columns, and the cell lysate was the subject of RNA isolation with miRNA Kit with RNeasy MinElute Columns (Qiagen).

### 4.8. Gene Expression Analysis of SH-SY5Y Cells and TSC Tissue Specimens

The Nanostring nCounter Neuroinflammation panel was used to measure gene expression in the SH-SY5Y cells and in tissue specimens. This assay measures gene expression for 770 genes associated with inflammation, immunity, neurobiology, metabolism, and stress. Nanostring assays were completed by the Research Technology Support Facility at Michigan State University. Each assay was initiated using 100 ng of RNA. Hybridization of the RNA to the report code set and capture probes was conducted at 65 °C for 17 h. After hybridization, the samples were then loaded on to the cartridge and then scanned at 555 FOV. Nanostring data were analyzed using nSolver software version 4.0. Pathway and network analysis was performed using STRING and DAVID [25,54]. Statistical analysis was performed in JMP v14.

## Figures and Tables

**Table 1:** TLR7/8-activating microRNAs are enriched in exosomes from epileptogenic tissue. Mature microRNA sequences are shown with highlighted G/U-rich motifs that activate TLR7/8. The microRNAs are sorted in order of fold change, comparing epileptogenic to non-epileptogenic exosomes.

microRNA	*p*-Value	FC	Sequence	UUGU	GUUU	UGUU	Total Motifs
hsa-miR-27a-5p	0.04	17.41	AGGGCUUAGCUGC**UUGU**GAGCA	1	0	0	1
hsa-miR-744-3p	0.03	5.49	C**UGUU**GCCACUAACCUCAACCU	0	0	1	1
hsa-miR-26a-2-3p	0.02	3.54	CCUAUUCUUGAUUAC**UUGUUU**C	1	1	1	3
hsa-miR-652-3p	0.04	3.54	AAUGGCGCCACUAGGG**UUGU**G	1	0	0	1
hsa-miR-21-5p	0.05	3.48	UAGCUUAUCAGACUGA**UGUU**GA	0	0	1	1
hsa-miR-142-3p	0.05	3.21	UGUAG**UGUUU**CCUACUUUAUGGA	0	1	1	2
hsa-miR-29b-1-5p	0.01	2.74	GCUG**GUUU**CAUAUGGUG**GUUU**AGA	0	2	0	2
hsa-miR-629-5p	0.03	2.48	UGG**GUUU**ACGUUGGGAGAACU	0	1	0	1
hsa-miR-28-3p	0.05	2.33	CACUAGA**UUGU**GAGCUCCUGGA	1	0	0	1
hsa-miR-3605-3p	0.01	1.90	CCUCCG**UGUU**ACCUGUCCUCUAG	0	0	1	1
hsa-miR-223-3p	0.04	1.75	UGUCA**GUUUGU**CAAAUACCCCA	1	1	0	2
hsa-miR-30a-3p	0.04	1.73	CUUUCAGUCGGA**UGUUU**GCAGC	0	1	1	2

## Data Availability

The data presented in this study are available on request from the corresponding author.
